# Altering Compliance of a Load Carriage Device in the Medial-Lateral Direction Reduces Peak Forces While Walking

**DOI:** 10.1038/s41598-018-32175-x

**Published:** 2018-09-13

**Authors:** Jean-Paul Martin, Qingguo Li

**Affiliations:** Bio-Mechatronics and Robotics Laboratory, Mechanical and Materials Engineering, Queen’s University, Kingston, K7L 3N6 Canada

## Abstract

Altering mechanical compliance in load carriage structures has shown to reduce metabolic cost and accelerative forces of carrying weight. Currently, modifications to load carriage structures have been primarily targeted at vertical motion of the carried mass. No study to date has investigated altering load carriage compliance in the medial-lateral direction. We developed a backpack specifically for allowing a carried mass to oscillate in the horizontal direction, giving us the unique opportunity to understand the effects of lateral mass motion on human gait. Previous modelling work has shown that walking economy can be improved through the interaction of a bipedal model with a laterally oscillating walking surface. To test whether a laterally oscillating mass can experimentally improve walking economy, we systematically varied step width above and below the preferred level and compared the effects of carrying an oscillating and fixed mass. Walking with an oscillating mass was found to reduce the accelerative forces of load carriage in both horizontal and vertical directions. However, load eccentricity caused the vertical force component to create a significant bending moment in the frontal plane. Walking with an oscillating mass led to an increase in the metabolic energy expenditure during walking and an increase in positive hip work during stance. The device’s ability to reduce forces experienced by the user, due to load carriage, holds promise. However, the requirement of additional metabolic energy to walk with the device requires future study to improve.

## Introduction

Load carriage has significant implications for how humans walk; carrying weight increases the metabolic energy required to walk^1–4^, increases muscle activity^2^, decreases stride length^5,6^, and increases double support time^6,7^. The additional demands of load carriage can lead to injuries such as stress fractures, metatarsalgia, lower back injuries, knee pain, and rucksack palsy^2,8^. How a load is carried has been shown to have a significant impact on energetic cost and muscle activity^9–11^. Therefore, improving load carriage structures has the potential to benefit users who interact often with heavy loads, be it industrial, military, or recreational environments.

Weight carried in a backpack undergoes similar accelerations to the trunk when walking. Altering mechanical compliance of a load carriage structure, such that the carried mass oscillates out-of-phase with the motions of the user in the vertical direction, can smooth the horizontal trajectory of the carried mass^12,13^. Out-of-phase oscillations can also reduce the peak accelerative forces of the carried weight that normally occur during step-to-step transitions. Alterations in compliance have successfully reduced peak vertical force across a variety of load carriage devices such as bamboo poles^12,14,15^, hand held weight^16^, and backpacks^13,17–19^. Two of these devices have also achieved reductions in the metabolic cost when compared to walking with a fixed load^13,14^.

During gait, the trunk not only oscillates in the vertical direction, but also moves medio-laterally with each step. Step width has been shown to modulate these oscillations, in addition to increasing both mechanical and metabolic energetics of walking^20^. Walking models have shown that the energetic cost of walking can be reduced through the interaction of an inverted pendulum walking model with a horizontally oscillating walking surface: where the walking surface assists gait by doing work on the walking model^21^. Additionally, lateral stability assistance, given by tethering subjects laterally to the lab environment using elastic elements, has shown to augment gait by decreasing the metabolic cost of walking^22–24^. This demonstrates that walking economy can be improved through a restorative force proportional to pelvis displacement from a global centreline. Both examples show that cyclical, well-timed external forces have the propensity to be beneficial for walking economy. No experimental work to date however has studied the effect of a medial-lateral oscillating mass on gait. We therefore propose altering the mechanical compliance of a load carriage device in the medial-lateral direction to study the mechanics and energetics of walking in the presence of an oscillating carried mass.

Changing how a carried load interacts with a user may have implications for frontal plane dynamics, including stability. Unlike sagittal plane dynamics, motion of the centre of mass in the frontal plane requires active stabilization by the central nervous system (CNS)^25^. The CNS actively stabilizes frontal plane motion by predicting future centre of mass position and altering foot placement to preserve a sufficient medial-lateral margin of stability^24,26–28^. The introduction of an additional degree-of-freedom, through the oscillation of a carried mass, could alter the body’s ability to predict centre of mass state; where the centre of mass state has been shown to be a predictor of step placement during locomotion^29,30^. In addition, humans are particularly sensitive to the application of non-steady state, random, perturbations during gait in the medial-lateral direction, as opposed to the fore-aft direction^31,32^. Sufficiently large perturbations can result in costly compensatory techniques, such as employing a lateral stepping strategy, which can be energetically costly for ambulation^22,26,28^. Therefore, the study of timing and magnitude of carried mass motion will help understanding of frontal plane dynamics during load carriage, and lead to the potential improvement of load carriage techniques.

**Figure 1:**
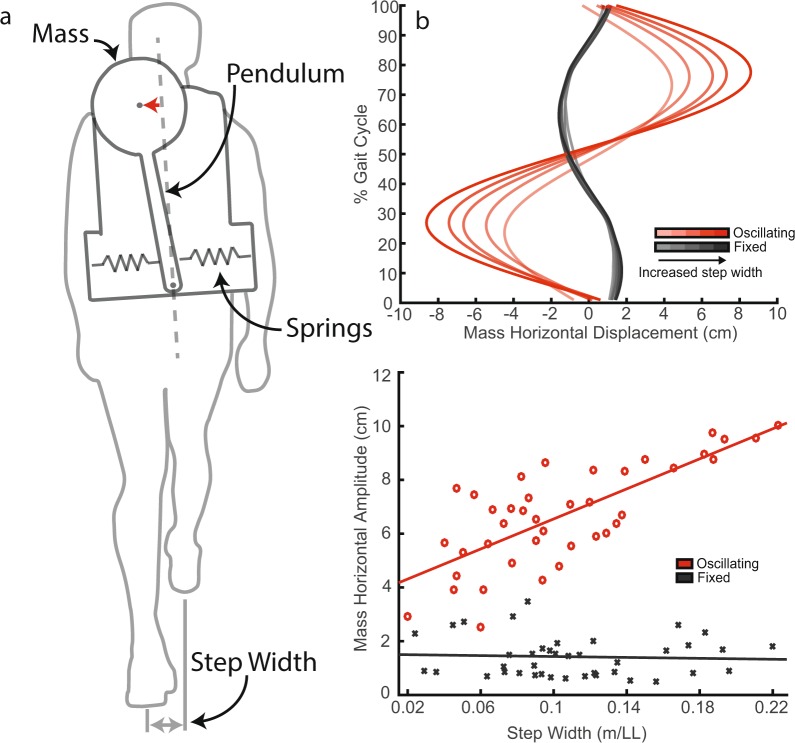
The load carriage device and carried mass response. (**a**) The load carriage device being worn by a subject. Horizontal displacement of the carried mass is indicated with a red arrow. Horizontal displacement is defined as the lateral component of mass displacement from the backpack’s local vertically directed axis (hatched line). (**b**) The carried mass’ horizontal displacement, with respect to the backpack’s vertically directed axis, as function of time. Increased transparency in the colour of the waveform indicates a reduction in step width. Waveforms represent the average across subjects for each enforced step width condition. (**c**) The carried mass’ average horizontal amplitude as a function of the measured step width. Each data point represents a subject’s average for the particular condition. The linear regression line shown for the oscillating condition has a slope of 27.9 ± 4.8 (mean ± SE) and offset of 3.8 ± 0.6. The linear regression line shown for the fixed condition has a slope of −0.8 ± 4.8 and offset of 1.51 ± 0.6.

We propose a device that suspends a carried mass, using a spring-loaded inverted pendulum, to gain insight to frontal plane dynamics during load carriage (Fig. 1a) (Supplementary Fig. S1). The inverted pendulum oscillates about small angles, creating horizontal displacement of the mass up to 10 cm. Trunk motion acts as an input to the single degree-of-freedom oscillator. When system components are chosen such that the natural frequency of the system is lower than the forcing frequency of the trunk, the mass oscillates out of phase with trunk motion (Fig. 1b).

The purpose of this study is to understand the effect of a laterally oscillating carried mass on a user’s energetics and mechanics. To investigate this, subjects walked with the device in unlocked (oscillating) and locked (fixed) states while carrying a 4.5 kg load. We then systematically altered step width, using biofeedback, above (+40%, +20%), below (−40%, −20%), and at (0%) preferred weighted walking step width (PSW). Step width was chosen to be systematically varied based on pilot testing (n = 5) that showed subjects preferred to reduce their step width by an average −18%, compared to their PSW during unlocked conditions. The following protocol allowed us to understand the device-user interaction at a range of step widths, as well as gain insight as to why subjects reduce their step width, when given free choice, when walking with an oscillating mass. Lastly, altering step width allows us to indirectly vary mass motion without having to change device parameters. As wider step widths lead to increased medial-lateral trunk motion, by systematically varying step width we alter the input motion to the backpack, thus increasing mass oscillation response. We hypothesize that an oscillating mass will reduce the peak medial-lateral interaction force between device and user, when compared to the fixed condition, due to out-of-phase oscillations. This, we hypothesize, will lead to reductions in mechanical work done by lower-limb joints. We further hypothesize that reduced joint work will lead to a decrease of the metabolic power of walking compared to the fixed condition.

## Results

### Device mechanics

As step width increased, the horizontal displacement of the carried mass significantly increased during oscillating conditions (Fig. 1c) (F(38) = 49.5, p = 2 · 10^−8^). Mass horizontal amplitude was significantly higher during oscillating conditions compared to fixed (F(1) = 526.0, p = 2 · 10^−36^). The increase in mass oscillation amplitude, from oscillating to fixed, showed significant interaction effects with step width: indicating a significantly greater increase at wider step widths compared to narrower step widths (F(1) = 39.3, p = 6 · 10^−8^).

Small amounts of mass displacement were reported during fixed conditions due to the overall compliance of the backpack frame and inverted pendulum, causing relative movement between the carried mass and load carriage frame. Mass horizontal displacement, for the fixed condition, did not significantly increase with step width (Fig. 1c) (F(38) = 0.1, p = 0.7).

**Figure 2:**
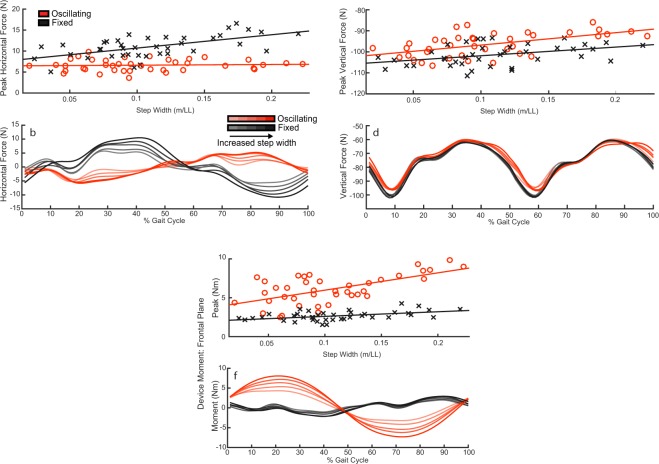
Device force and moment. (**a**) Peak medial-lateral interaction force between device and user as a function of step width. Each data point represents a subject’s average for the particular condition. (Regression coefficients: Oscillating, slope = 1.7 ± 8.2 (mean ± SE), offset = 6.5 ± 1.0. Fixed, slope = 31.8 ± 8.2, offset = 7.5 ± 1.0). (**b**) Medial-lateral interaction force between device and user as a function of % gait cycle. Increased transparency in the colour of the waveform indicates a reduction in step width. Waveforms represent the average across subjects for each enforced step width condition. (**c**) Peak vertical interaction force between device and user as a function of step width. (Regression coefficients: Oscillating, slope = 59.3 ± 21.3, offset = −102.7 ± 2.5. Fixed: slope = 41.5 ± 21.3, offset = 106.0 ± 2.5). (**d**) Vertical interaction force between device and user as a function of % gait cycle. (**e**) Peak device moment in the frontal plane as a function of step width. (Regression coefficients: Oscillating, slope = 22.2 ± 5.1, offset = 3.7 ± 0.6. Fixed, slope = 5.9 ± 5.1, offset = 2.0 ± 0.6). (**f**) Frontal plane moment as a function of % gait cycle.

When oscillating, the peak medial-lateral interaction force between device and user significantly decreased, compared to fixed conditions (Fig. 2a) (F(1) = 117.4, p = 4 · 10^−17^). This decrease in peak medial-lateral interaction force between conditions became significantly greater as step width became wider (F(1) = 13.5, p = 0.0004). Peak medial-lateral interaction force timing, across all step width conditions, was significantly out of phase with respect to fixed load conditions by a phase angle of 165 ± 36° (Fig. 2b) (t(39) = −34.2, p = 1 · 10^−30^).

The magnitude of the peak vertical interaction force, between device and user, significantly decreased with an oscillating mass, compared to a fixed mass (Fig. 2c) (F(1) = 25.1, p = 3 · 10^−6^). The decrease in magnitude of peak vertical interaction force did not significantly differ across step width conditions (F(1) = 0.7, p = 0.4). No change in loading phase was observed in the vertical direction.

Although decreases in peak horizontal and vertical interaction force were observed, the device’s frontal plane moment, acting on the user’s trunk, significantly increased when the mass was oscillating (Fig. 2e) (F(1) = 197.1, p = 8 · 10^−23^). Significant interaction effects between backpack condition (oscillating/fixed) and step width were observed (F(1) = 10.2, p = 0.002): where the increase in frontal plane moment in the oscillating condition, compared to fixed, increased with wider step width.

### Metabolic expenditure

**Figure 3:**
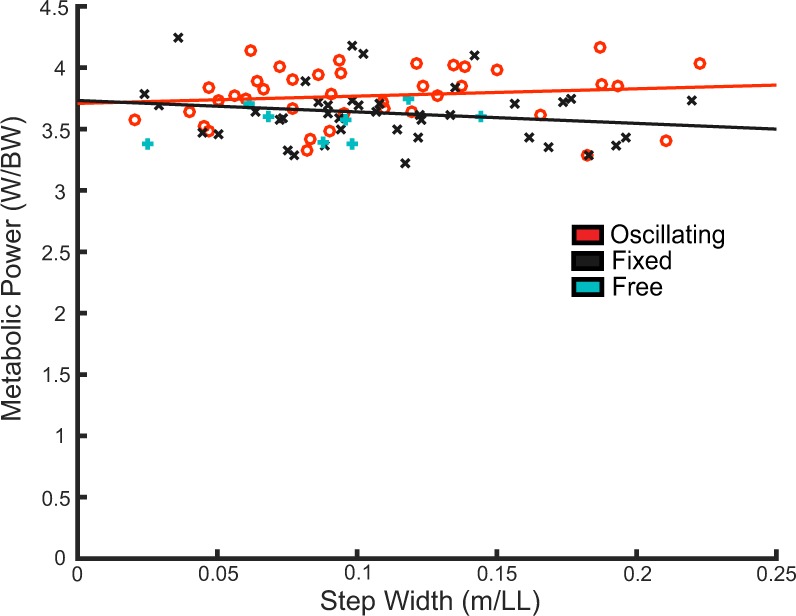
The average metabolic power during walking as a function of step width. The free condition indicates the metabolic cost of walking under the free step width condition (average free step width = −22.4 ± 11.4% PSW (mean ± SD)). Each data point represents a subject’s average for the particular condition. (Regression coefficients: Oscillating, slope = 0.6 ± 1.1 (mean ± SE), offset = 3.7 ± 0.1. Fixed: slope = −0.9 ± 1.1, offset = 3.7 ± 0.1).

The metabolic cost of walking significantly increased when the carried mass was oscillating, compared to fixed condition (Fig. 3) (F(1) = 7.3, p = 0.009). The metabolic cost of walking did not significantly vary with step width (F(1) = 0.05, p = 0.8). There were no significant interaction effects between backpack condition and step width (F(1) = 2.04, p = 0.2). In addition to the first order model shown in Fig. 3, a second order model describing metabolic cost as function of step width was also conducted. However, the second order coefficient was found to not be significantly different than zero (t(65) = 1.2, p = 0.2).

Under the free step width condition (no step width enforcement, mass oscillating) subjects chose to walk at an average −22.4 ± 11.4% (mean ± SD) of their PSW at a metabolic cost of 3.6 ± 0.1 W/BW (0.18 ± 0.01 ml O2/s/BW). Compared to the closest enforced step width and oscillating condition (−20% PSW), there was no significant difference in step width (t(7) = −0.3, p = 0.7). The metabolic cost of walking during the −20% PSW was 3.9 ± 0.1 W/BW (0.19 ± 0.01 ml O2/s/BW). Therefore, the free choice of step width resulted in a significant decrease of 8 ± 6% in metabolic cost compared to the step width enforced condition (t(7) = 4.5, p = 0.003).

### Lower-limb mechanics

**Figure 4:**
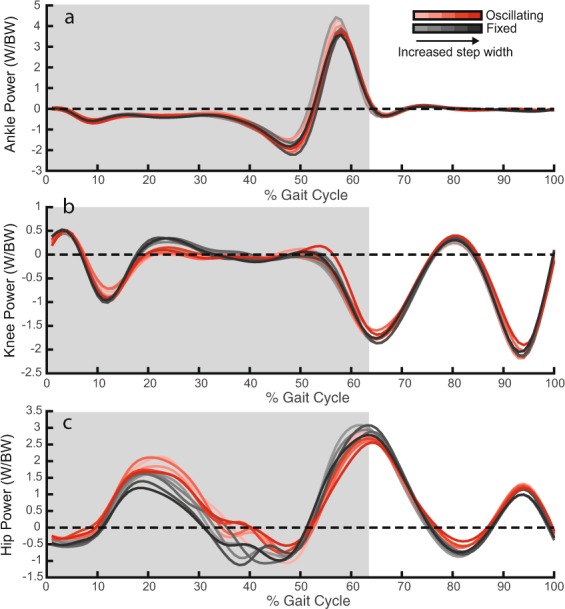
Ankle, knee, and hip joint power. (**a**) Ankle joint power as a function of % gait cycle. (**b**) Knee joint power as a function of % gait cycle. (**c**) Hip joint power as a function of % gait cycle. Increased transparency in the colour of the waveform indicates a reduction in step width. Grey background shading indicates a region for which joint work is calculated (stance). Waveforms represent the average across subjects for each enforced step width condition.

Joint power at the ankle, knee, and hip are shown in Fig. 4. Significant increases in positive work done by the hip during stance were observed when walking with an oscillating mass, compared to a fixed mass (Table 1) (F(1) = 10.9, p = 0.001). A significant interaction effect between backpack condition and step width indicated that the additional positive hip work performed during oscillating conditions increased at wider step width (F(1) = 5.6, p = 0.02). Increases in positive hip work were accompanied by a significant increase, compared to fixed, in peak positive hip power in early stance (F(1) = 22.7, p = 9 · 10^−6^). However, there was no significant interaction effects between step width and backpack condition on positive hip work (F(1) = 2.7, p = 0.1).

**Table 1:** Regression coefficients for positive and negative joint work performed by the ankle, knee, and hip during the stance phase.

Joint	Backpack Condition		Positive and Negative Work	
			Slope [J/BW * SW] (mean ± SE)	Offset [J/BW] (mean ± SE)
Ankle	Fixed	Pos.	0.15 ± 0.46	0.29 ± 0.05
		Neg.	−0.14 ± 0.83	−0.34 ± 0.10
	Oscillating	Pos.	0.07 ± 0.46	0.30 ± 0.05
		Neg.	0.06 ± 0.83	−0.35 ± 0.10
Knee	Fixed	Pos.	−0.12 ± 0.36	0.15 ± 0.04
		Neg.	−0.13 ± 0.44	−0.25 ± 0.05
	Oscillating	Pos.	−0.17 ± 0.36	0.13 ± 0.04
		Neg.	−0.14 ± 0.44	−0.25 ± 0.05
Hip	Fixed	Pos.	−0.39 ± 0.44*	0.57 ± 0.05*
		Neg.	−0.12 ± 0.44*	−0.19 ± 0.05*
	Oscillating	Pos.	0.65 ± 0.44*	0.53 ± 0.05*
		Neg.	0.48 ± 0.44*	−0.18 ± 0.05*

Significant differences between backpack conditions (fixed-oscillating) are indicated by an asterisks (*α* < 0.05).

Negative work done by the hip during stance significantly decreased when walking with an oscillating mass, compared to fixed (F(1) = 11.6, p = 0.001). There were no significant interaction effects between step width and backpack condition for negative hip work performed (F(1) = 1.9, p = 0.2).

Backpack condition had no significant effect on positive work done by the knee (F(1) = 1.6, p = 0.2) or ankle (F(1) = 0.06, p = 0.8), or negative work done by the knee (F(1) = 0.003, p = 0.9) or ankle (F(1) = 0.04, p = 0.8) during stance. Additionally, step width condition had no effect on the positive (F(1) = 0.7, p = 0.4) and negative (F(1) = 0.4, p = 0.5) knee work, or positive (F(1) = 0.2, p = 0.6) and negative (F(1) = 0.01, p = 0.9) ankle work done during stance.

### Spatial-temporal gait parameters

**Figure 5:**
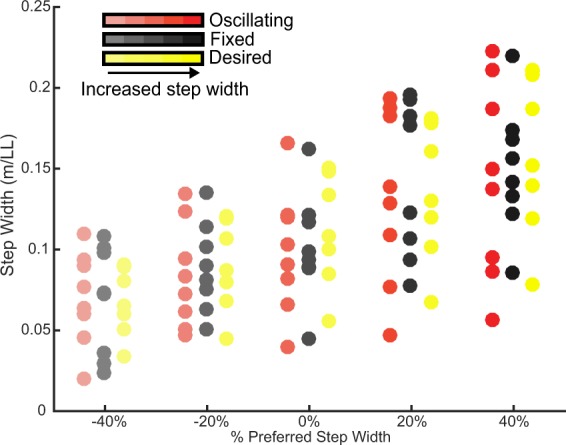
Step width adherence. Subject step width adherence levels over all enforced step width conditions. Desired step width was the step width displayed on the biofeedback screen. Each data point represents a subject’s average for the particular condition.

Using a display to feedback subject’s step width at heel strike, step width was manipulated to desired percentages of the subject’s preferred weighted walking step width (Fig. 5a). Adherence error during oscillating conditions were 3 ± 7% (mean ± SD), 10 ± 6%, 14 ± 9%, 4 ± 10%, and 13 ± 9% for −40%, −20%, 0%, 20%, and 40% PSW. For the fixed backpack condition, adherence errors in enforced step width were 8 ± 6%, 2 ± 6%, 11 ± 7%, 5 ± 9%, and 5 ± 6% for −40%, −20%, 0%, 20%, 40% PSW.

No significant changes in step length, stride frequency, and step width variability were observed between backpack conditions (F(1) = 0.4, p = 0.5; F(1) = 0.9, p = 0.3; F(1) = 0.03, p = 0.9) and between step width conditions (F(1) = 0.2, p = 0.6; F(1) = 0.1, p = 0.7; F(1) = 0.2, p = 0.6).

## Discussion

In this study, we suspended a mass using an inverted pendulum, altering the compliance between carried load and load carriage structure in the medial-lateral direction. Previous load carriage structures, with altered vertical compliance, have shown decreases in peak vertical interaction force^12–16,18^ and the metabolic cost of walking^13,14^. As hypothesized, we similarly observed reductions in peak medial-lateral interaction force, between device and user. There was also an associative phase shift in the peak force timing, where the medial-lateral interaction force began to act like a restorative force on the body: providing a force on the trunk of the user roughly corresponding to the opposite direction the trunk was translating. However, the device caused an increase in the metabolic power during walking. Although a significant reduction in peak medial-lateral interaction force was observed, walking with an oscillating mass resulted in a significant increase in the interaction moment between device and user in the frontal plane. If we consider the frontal plane moment to be the sum of the medial-lateral force due to the carried mass (*F*_*m*_, *x*) multiplied by the vertical distance of the mass to the load cell (*d*_*y*_), plus the vertical force due to the carried mass multiplied (*F*_*m*_, *y*) by the mass medial-lateral displacement (*d*_*x*_): as the medial-lateral displacement increases, the greater in magnitude vertical force component, *F*_*y*_, will create a significant bending moment about the load cell connection point (Free body diagram: Supplementary Fig. S2). The bending moment about the load cell connection point will be transferred through the rigid backpack frame and applied to the user’s trunk through a series of load carriage connection points (waist and shoulder straps). Current results do, however, indicate that a potential device operating region that reduces interaction loads experienced by the user could exist at lower oscillation amplitudes that also minimizes the frontal plane interaction moment. The reduction in vertical interaction force, between device and user, of the carried mass was observed across all oscillation amplitudes. Although the reduction in medial-lateral interaction force was minimized at narrow step widths, the bending moment due to mass eccentricity was also reduced at smaller oscillation amplitudes. Therefore, a state in which the beneficial interaction force reduction coincides with minimal bending moment may exist in regions of smaller oscillation amplitudes.

Previous work has shown that joint work and metabolic energy expenditure increases approximately linearly with carried load^4^. We estimated joint work done during stance phase, as previous work has shown the redirection of the COM trajectory in the M-L direction to occur during this phase of gait^20,33^. As our device alters the timing of which the lower-limbs redirect the carried mass, we expected greatest change to occur during stance phase. While walking with an oscillating mass at wider step widths, the peak power and positive work done by the hip during stance significantly increased. The increase in positive hip power during stance was accompanied by a tendency towards greater hip moment in the frontal plane during early to terminal stance (Supplementary Fig. S3). Contrary to the positive work performed by the hip, negative work done by the hip was found to significantly decrease during stance phase when walking with an oscillating mass. The decrease in negative work done was accompanied by a tendency towards decreased hip moment, in the sagittal plane, during mid to terminal stance (Supplementary Fig. S3). As muscles perform positive work with an observed efficiency of approximately 25%, and negative work is performed at an efficiency of −120%^34,35^, we suspect that the reductions in negative work done by the hip contribute relatively small energetic savings in comparison to the increase in positive work performed by the hip. However, this analysis does not account for passive mechanisms that may store and return energy during negative and positive work phases.

Subjects, when given the free choice to select step width, reduced their average step width by −22 ± 11.4% PSW when walking with the oscillating mass. This is consistent with a pilot study (n = 5) that showed subjects preferred to reduce their step width by an average of −18% compared to their PSW when walking with an oscillating mass. It is unknown however why subjects consistently select a step width more narrow then preferred levels during fixed conditions. In the field of locomotion, walking behaviour is thought to be governed by the minimization of metabolic energy^36–39^. In the present study, a metabolic power landscape was mapped from −40% PSW to 40% PSW. However, a limitation to our experiment is that we did not have the statistical power to discern local minima in the metabolic power cost landscape. Metabolic power has been shown in previous work to be a second order relationship with respect to step width^20^. We likewise initially assumed a second order model for metabolic power as a function of step width. Statistical tests revealed that the coefficient for higher order terms were not significantly different then zero. This means that the measurement variability exceeds the systematic changes in step width, which may be the reason why we observe negative higher order coefficients in both first and second order models: the negative higher order coefficient is due to measurement noise, not a systematic change in step width. Therefore, based on the current evidence, we are unable to determine whether subjects chose a step width that considered metabolic power as the primary cost function, or minimized some other cost function. Future experiments should be conducted, perhaps with additional subjects, to give insight whether subjects adopt novel gait patterns with our device that are dictated by metabolic costs^36^, mechanical costs^40^, stability^41^, comfort^42^, or some combination of the above^40,43^.

A limitation of the study was the inability to separate step width effects from mass amplitude effects during oscillating conditions. A fixed mass condition was used as a baseline for each enforced step width condition to account for step width effects. For statistical analysis, step width and backpack condition are assumed to be independent.

The biofeedback methods used in this study resulted in a max step width adherence error of 13 ± 9% with an average of 7 ± 6% across all conditions. An associative metabolic cost due to step width enforcement was observed, and assumed to effect all conditions uniformly. Although no direct comparison exists, the free condition occurred at a step width of −22.4 ± 11.4% of the PWS and exhibited a metabolic cost of 3.6 ± 0.1 W/BW. This was a decrease of 7.9 ± 5.9% compared to the average metabolic cost for the enforced −20% step width condition. This is consistent with previous studies that observed a 14% increase in metabolic cost for both step width and frequency control^22^, and a 8% increase for enforcing projected step locations^44^.

In summary, we altered a load carriage device such that the carried mass oscillated in the medial lateral direction. Our motivation was to study the effect of timing and magnitude of carried mass motion on gait kinetics and metabolic power during load carriage. We found that although peak interaction forces of the carried mass were reduced in both horizontal and vertical directions, load eccentricity created a large bending moment in the frontal plane. Results indicated that potential assistive operating regions for the device may exist around lower oscillation amplitudes. These results contribute to the understanding of load carriage and gait dynamics, as well they have the potential to lead to improvements in load carriage techniques.

## Methods

### Medial-lateral oscillating load carriage device

The load carriage device consists of a mass (weight = 4.5 kg), suspended by an inverted pendulum, attached to a rigid backpack frame worn by the user (Fig. 1a and Supplementary Fig. S1). The inverted pendulum was set to a length of 30 cm, with the option of 5 linear springs acting at 2 cm increments up the pendulum, providing a restorative force back to the centreline. Spring constants were chosen such that the ratio of the device’s natural frequency was 0.7 of the subject’s characteristic forcing frequency, determined during acclimatization while walking with the device in a fixed condition. The natural frequency of the device was defined as the undamped natural frequency of the carried mass, inverted pendulum, and springs. The torsional spring constant was calculated using a spring, mass, damper single degree of freedom model. A custom Matlab script (Mathworks Inc., MA, USA) was used to convert the torsional spring constant to an equivalent configuration of parallel linear springs. The model’s ability to determine spring configuration based on a desired natural frequency was confirmed by mounting the device to a rigid test apparatus and recording the pendulum’s free vibration response to initial conditions. The device’s mechanical damping constant was found to be 0.11 *Nm*/*rad* · *s*^−1^. Pendulum and springs were housed in a rectangular frame weighing 2.2 kg. The frame was mounted to a rigid backpack frame (weight = 2.4 kg) via a 6 degree-of-freedom load cell (Mini-45, ATI Industrial Automation, Apex, NC) (weight = 92 g). During fixed conditions, the pendulum was fixed via a bolt through the pendulum shaft, threading in to the device’s frame.

### Experimental protocol

Eight subjects (n = 8) walked at 1.25 m/s with the device harbouring an oscillating or fixed mass at 5 varying step widths. All subjects were healthy, young adults, with no known prior gait pathologies, and provided informed consent (age = 24.5 ± 2.9 years, height = 176 ± 5 cm, weight = 68.9 ± 7.7 kg, leg length (floor-GT) = 91 ± 2 cm, males = 5, females = 3). The experiment was approved by the General Research Ethics Board (GREB) of Queen’s University. All methods performed were in accordance with GREB’s approved guidelines.

Subjects familiarized themselves with the split belt treadmill (AMTI Force-Sensing Tandem Treadmill, AMTI Inc., MA) and device during an acclimatization session 24–48 hours prior to the data collection. During the acclimatization, subjects walked with the device in the fixed condition for 10 min, for which the last 2 min were used to obtain preferred step frequency (characteristic forcing frequency), and their preferred step width, to be used as their PSW during experimentation. Subjects then walked with the device in an oscillating condition with no step width enforcement (5 min), 0% preferred SW (5 min), −20% SW (2 min), 20% SW (2 min), −40% SW (2 min), and 40% SW (2 min).

On experimental day, subjects performed a 6 min warm-up, where 3 min consisted of walking with the device in an oscillating condition, and 3 min in an oscillating condition with 0% preferred SW enforcement. We then tested 5 step width conditions (−40%, −20%, 0%, 20%, 40% of PSW) while a carried mass of 4.5 kg was either oscillating or fixed on a backpack. One additional oscillating condition was added where the subject was free to walk with no step width enforcement. All conditions were randomized in order and 6 min in duration. Kinematic, kinetic, metabolic data, and device mechanics were collected in the last 2 min of every trial after 4 min of walking, to ensure steady state metabolic measurements. A 6 min quiet standing trial was performed beforehand to determine each subject’s resting metabolic rate.

**Figure 6:**
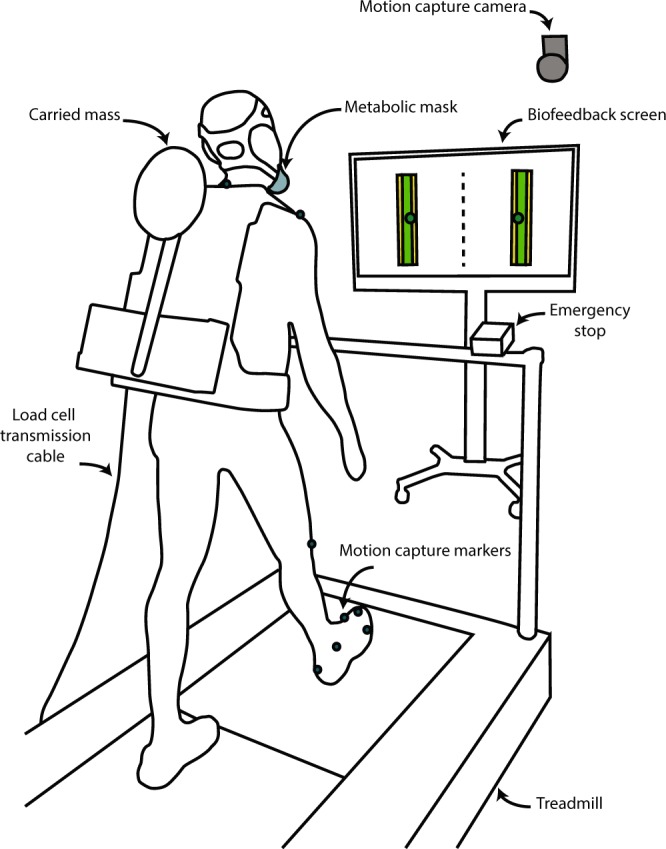
Schematic of the experimental setup. The carried mass was suspended via an inverted pendulum, mounted to a backpack frame, worn by the user. A cable transmitted load cell force and moment data in 6DOF. A respirometry mask worn by the user collected expired gas. Motion capture markers collected subject kinematics. A split belt treadmill collected ground reaction force and moment data. Step width was shown to the subject on a screen via biofeedback to enforce step width within a target range.

Step width enforcement was provided via biofeedback using a television screen to display the subject’s step width (Fig. 6). Calcaneus marker location and force plate data were streamed in real time to a custom Matlab script using Qualisys’ Matlab Client (Qualysis, Sweden). At the onset of heel strike, as determined by a force threshold (20 N) being exceeded in the vertical component of the ground reaction force, the horizontal distance between calcaneus markers were displayed. Feedback was given where a registered step width in the passband (±10% of the desired step width) was denoted green. Two passbands were displayed for each foot on the screen, an acceptable passband shaded yellow (±10% of the desired step width), and a desired passband shaded green (±5% of the desired step width).

### Data Analysis

#### Device mechanics

Device force and moment were measured using a 6DOF load cell mounted between the device and backpack frame sampling at 500 Hz and passed through a 4th order low-pass zero-phase filter with a cutoff frequency of 8 Hz. Peak horizontal and vertical force were defined as the maximum magnitude of force over each stride, averaged over the last 2 min of each condition. The phase angle of peak medial-lateral force was found by performing a discrete Fast Fourier Transform over the last 2 min of each condition. The average phase angle between fixed and oscillation conditions, across all subjects, was found as the difference between the phase angle of each oscillating condition and the subject’s fixed condition and then averaged across all conditions and subjects. The interaction moment, between device and user, in the frontal plane was defined as the moment measured about the load cell’s anteriorly directed axis. The interaction force measured at the device’s load cell was assumed to be the interaction force between the device and user. Forces due to the acceleration of the backpack frame and compliance of the backpack straps were not accounted for.

Pendulum angle was estimated using motion capture (Oqus, Qualysis, Sweden) and was defined as the angle between a frame mounted coordinate system and a pendulum mounted coordinate system. The backpack frame mounted coordinate system was defined by 5 markers: two located on the left side of the frame defining a superiorly directed vector, two on the front defining a laterally directed vector, and one on the pendulum origin. A pendulum mounted coordinate system was defined by a custom four marker cluster attached to the pendulum shaft. Motion capture sampled at 100 Hz and pendulum angle was filtered using 4th order low-pass zero-phase filter with a 8 Hz cutoff frequency. Horizontal mass motion was defined as the lateral component of mass displacement from the backpack’s local vertically directed axis.

#### Metabolic cost

Metabolic power was estimated through the measure of volume flow rate of oxygen consumption (VO2) and carbon dioxide production (VCO2) with an open respirometry unit (K4b2, Cosmed, Italy). Metabolic power in Watts was calculated using the standard equation from Brockway^45^. The last two minutes of each condition were averaged to determine the gross metabolic power. The net metabolic power was determined by subtracting subject’s quiet standing resting metabolic rate then normalizing to body weight.

#### Lower-limb mechanics

Lower limb joint power of the right leg was estimated using three dimensional inverse dynamics with a custom Matlab script, using the methods described in^46^. Segment kinematics of the right foot, shank, thigh, and pelvis were estimated using motion capture, sampled at 100 Hz, tracking reflective markers placed on bony landmarks of lower limbs (2^nd^ order low-pass zero-phase filtered at a 8 Hz cutoff). Marker placement and definitions of segment local coordinate systems were defined using a modified method from^46^. The modification was required as the backpack’s waist straps covered the anterior and posterior superior iliac spine bony landmarks used in defining the pelvis coordinate system. We assumed that the trunk and pelvis were a single rigid body segment, and tracked pelvis motion using a trunk segment coordinate system (C7, sternal notch, and right and left acromion markers). The transformation matrix relating the trunk coordinate system and the pelvis coordinate system was recorded in a static pose before testing. Pelvis markers were then removed before placing the backpack on the subject. Then, the pelvis was tracked using the trunk segment’s coordinate system for subsequent walking trials. The ground reaction force was measured using a split belt treadmill sampling at 500 Hz. Joint kinetics were filtered using a 2nd order low-pass zero-phase filter with 8 Hz cutoff, and three dimensional ground reaction forces were filtered at a 4th order low-pass zero-phase filter with 40 Hz cutoff. Body segment mass and radius of gyration were determined using anthropometric data from^47^.

#### Spatial-temporal gait parameters

Step width and length were determined at heel strike as the horizontal and for-aft distance between calcaneus markers. Step width variability was defined as the standard deviation of step width over the 2 min collection period.

#### Statistical tests

Statistical tests were performed in Matlab for mass motion, device interaction force and moment, metabolic expenditure, joint peak power and work, step width, step width variability, step length, and stride frequency. An analysis of covariance model was applied to the aforementioned gait and device variables, with oscillating and fixed backpack conditions as treatment levels, subject as a blocking variable, and step width as a covariate (*α* < 0.05). Pairwise comparisons were made using a repeated measures t-test (loading phase) (*α* < 0.05). A second order least-squares regression was conducted on metabolic expenditure, with step width evaluated as the predictor. Coefficients of the model were tested whether they were significantly different than zero using paired t-tests (*α* < 0.05).

## Electronic supplementary material

Supplementary Information

## Data Availability

The datasets generated during the current study are available from the corresponding author on request.
