# Supplementary material for: Altering Compliance of a Load Carriage Device in the Medial-Lateral Direction Reduces Peak Forces While Walking
Source: Sci Rep. 2018 Sep 13;8:13775. doi: 10.1038/s41598-018-32175-x (PMC6137106; doi:10.1038/s41598-018-32175-x)
Supplement: Supplementary file 1 — Supplementary Information [file 41598_2018_32175_MOESM1_ESM.pdf]

Supplementary Materials: Altering Compliance of a Load Carriage Device in the Medial-Lateral Direction Reduces Peak Forces While Walking

Jean-Paul Martin[1] **and Qingguo Li**[1,*]

[1]Bio-Mechatronics and Robotics Laboratory, Mechanical and Materials Engineering, Kingston, K7L 3N6, Canada
[*]ql3@queensu.ca

Load Carriage Device

The fabricated device is a simple inverted pendulum housed in a rectangular frame (weight = 2.2 kg) (Fig. S1a). The device is then mounted on to a pre-existing backpack frame (weight = 2.4 kg) which is worn by the user (Fig. S1b). A 6 degree-of-freedom load cell (Mini-45, ATI Industrial Automation, Apex, NC)(weight = 92 g) is mounted between the device frame and the backpack frame. The pendulum arm itself is a rectangular shaft of 80-20 Aluminum. The mass carried is then clamped on to the pendulum shaft at various lengths (12.5-40cm). The mass carried is simulated using free weights in increments of 4.5kg. Linear springs, acting at various insertion points (2-10cm) up the pendulum's shaft provide an effective torsional spring constant up to 250 Nm/rad. Two spring anchor locations are located at each increment of 2cm. Matching springs are attached in the opposing direction, with initial deflections equal to zero when the pendulum angle is at its maximum ($\pm 20^o$). Maximum pendulum angle is defined as the maximum stroke of commercially available linear springs.

A simplified schematic of the device in Fig. S2a illustrates force components and moment arms that contribute to frontal plane device interaction moment (M_{lc} in figure). A secondary side view (Fig. S2b) shows the load pathways from the device, through the load cell, to the backpack frame.

Lower-Limb Joint Moment

Joint moments of the ankle, knee, and hip in the sagittal and frontal planes (Fig. S3).

Lower-Limb Joint Angles

Joint angles of the ankle, knee, and hip in the sagittal and frontal planes (Fig. S4).

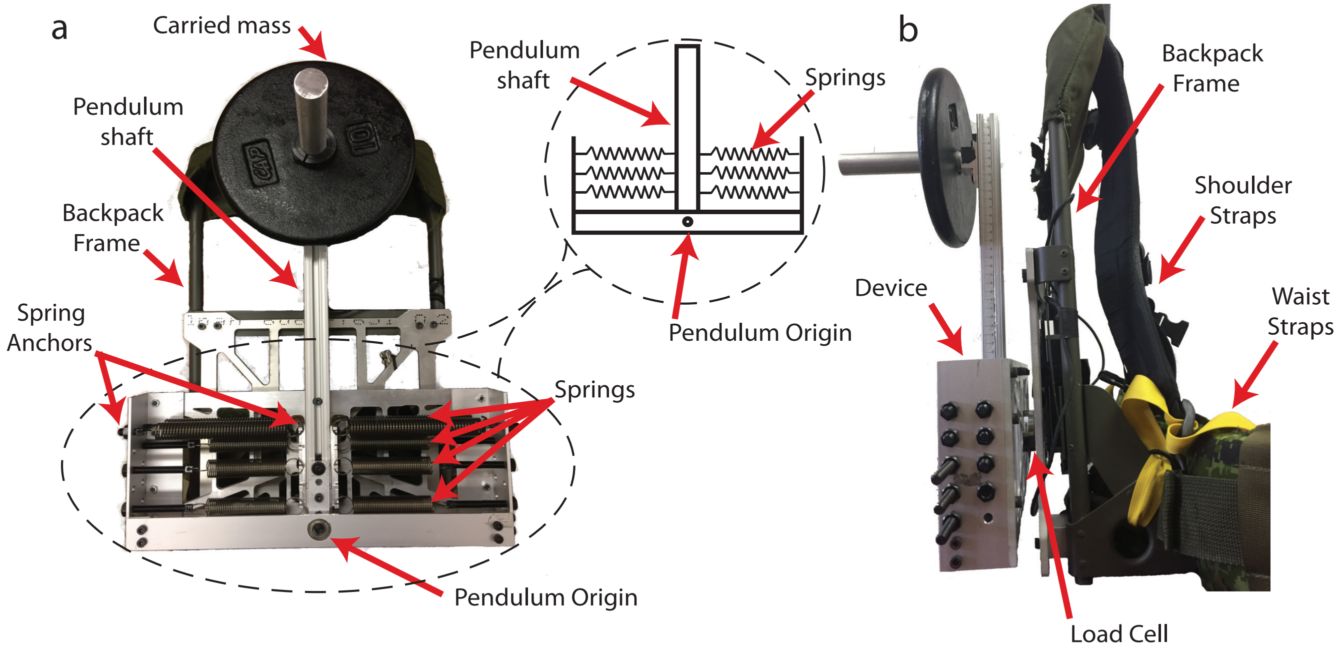

Figure S1. Load carriage device. (a) Front view of the load carriage device. Blown up area shows simplified diagram of how linear springs act up the length of the pendulum. (b) Side view of the load carriage device. Backpack shoulder straps and waist straps are included to show entirety of device.

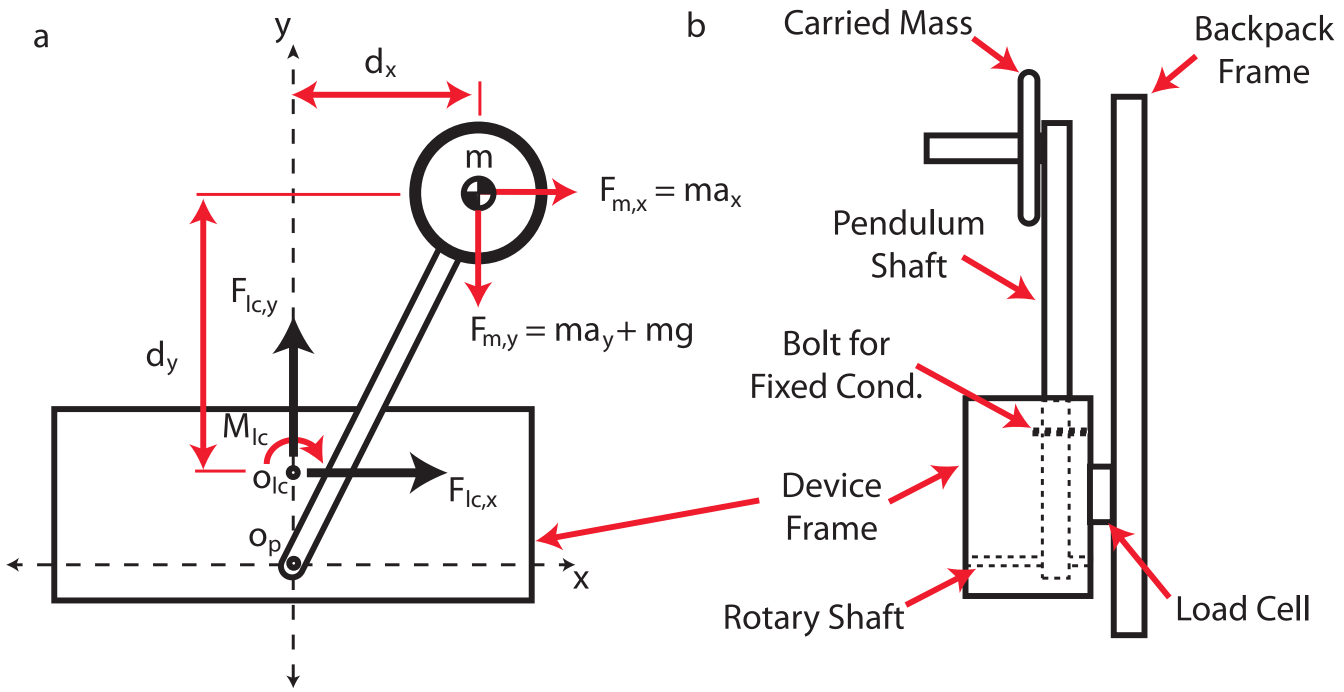

Figure S2. Device schematics. (a) Front view of the device. The carried mass m, oscillates on an inverted pendulum with origin o_p. The carried mass has a horizontal force component, $F_{m,x}$, equal to the product of carried mass and its acceleration in the x direction, a_x. The vertical force component, $F_{m,y}$, is equal to the mass multiplied by the acceleration of the mass in the y direction, a_x, and the product of the mass and acceleration due to gravity. The moment about the load cell's for-aft axis, located at the load cell origin o_{lc}, is equal to the $F_{m,x} \cdot d_y + F_{m,y} \cdot d_x$, where d_y and d_x are the distance from the centre of the carried mass to the load cell in the vertical and horizontal direction. Interaction forces of the device, acting on the user, are shown as $F_{lc,x}$ and $F_{lc,y}$. (b) A side view of the device indicating load pathways from the device, through the load cell, on to the backpack frame.

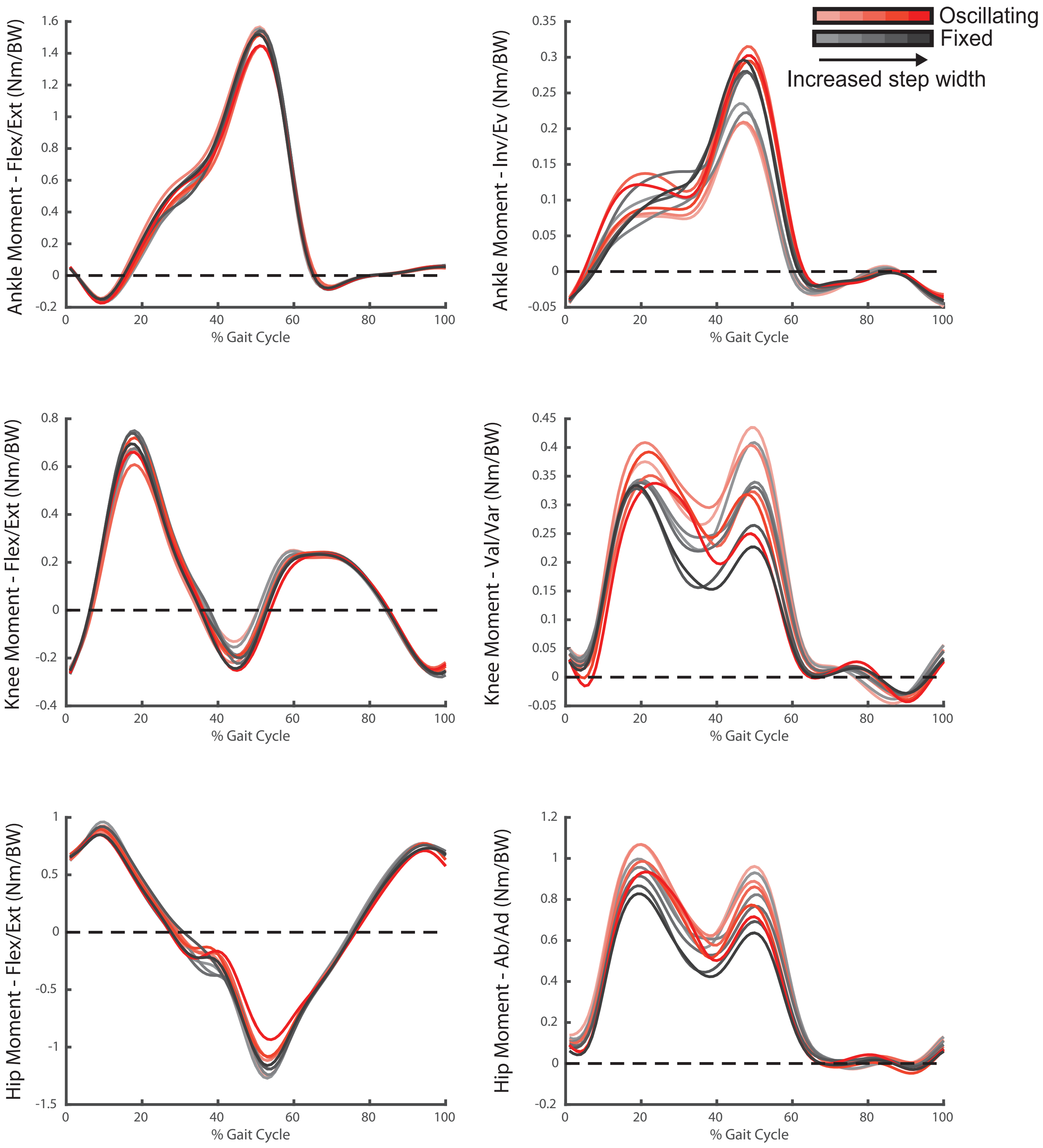

Figure S3. Joint moment for ankle, knee and hip in the sagittal and frontal planes, averaged across participants. Increased waveform transparency indicates a decrease in step width. Values of step width are -40%, -20%, 0%, 20%, 40% preferred weighted walking step width.

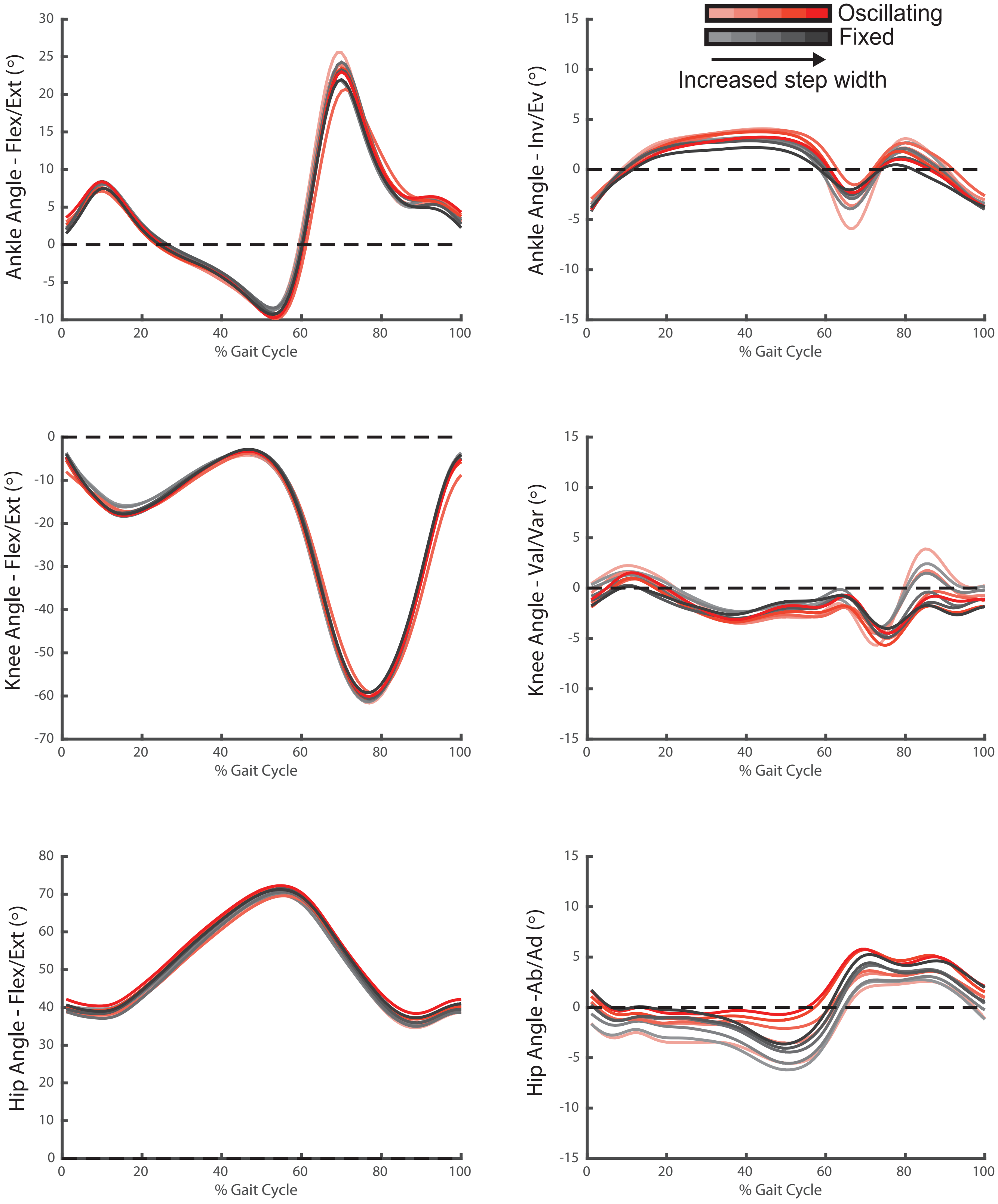

Figure S4. Joint angles for ankle, knee and hip in the sagittal and frontal planes, averaged across participants. Increased waveform transparency indicates a decrease in step width. Values of step width are -40%, -20%, 0%, 20%, 40% preferred weighted walking step width.
